# Exercise-Induced Browning of White Adipose Tissue and Improving Skeletal Muscle Insulin Sensitivity in Obese/Non-obese Growing Mice: Do Not Neglect Exosomal miR-27a

**DOI:** 10.3389/fnut.2022.940673

**Published:** 2022-06-17

**Authors:** Dongxue Wang, Xihuan Zhang, Yibai Li, Lihong Jia, Lingling Zhai, Wei Wei, Li Zhang, Hongkun Jiang, Yinglong Bai

**Affiliations:** ^1^Department of Maternal and Child Health, School of Public Health, China Medical University, Shenyang, China; ^2^The Second People’s Hospital of Jiashan, Jiaxing, China; ^3^Xinzhou District Center for Disease Control and Prevention, Wuhan, China; ^4^The First Division of Clinical Medicine, China Medical University, Shenyang, China; ^5^Department of Dermatology, First Hospital of China Medical University, Shenyang, China; ^6^Key Laboratory of Immunodermatology, Ministry of Education and NHC, National Joint Engineering Research Center for Theranostics of Immunological Skin Diseases, Shenyang, China; ^7^Department of Pediatrics, First Hospital of China Medical University, Shenyang, China

**Keywords:** exercise, exosomes, miR-27a, browning effect of white adipose tissue, skeletal muscle, insulin sensitivity

## Abstract

Exercise is considered as a favorable measure to prevent and treat childhood obesity. However, the underlying mechanisms of exercise-induced beneficial effects and the difference between obese and non-obese individuals are largely unclear. Recently, miR-27a is recognized as a central upstream regulator of proliferator-activated receptor γ (PPAR-γ) in contributing to various physiological and pathological processes. This study aims to explore the possible cause of exercise affecting white adipose tissue (WAT) browning and reversing skeletal muscle insulin resistance in obese/non-obese immature bodies. For simulating the process of childhood obesity, juvenile mice were fed with a basal diet or high-fat diet (HFD) and took 1 or 2 h swimming exercise simultaneously for 10 weeks. The obese animal model was induced by the HFD. We found that exercise hindered HFD-induced body fat development in growing mice. Exercise modified glucolipid metabolism parameters differently in the obese/non-obese groups, and the changes of the 2 h exercise mice were not consistent with the 1 h exercise mice. The level of serum exosomal miR-27a in the non-exercise obese group was increased obviously, which was reduced in the exercise obese groups. Results from bioinformatics analysis and dual-luciferase reporter assay showed that miR-27a targeted PPAR-γ. Exercise stimulated WAT browning; however, the response of obese WAT lagged behind normal WAT. In the HFD-fed mice, 2 h exercise activated the IRS-1/Akt/GLUT-4 signaling pathway in the skeletal muscles. In summary, our findings confirmed that exercise-induced beneficial effects are associated with exercise duration, and the response of obese and non-obese bodies is different. Exosomal miR-27a might be a crucial node for the process of exercise-induced browning of WAT and improving skeletal muscle insulin sensitivity.

## Introduction

Childhood obesity has become one of the most serious global public health challenges in the 21st century, which is an independent disease and can lead to various health impairments, such as insulin resistance, dysglycemia, hypertension, fatty liver, and psychosocial complications (1, 2). Irregular or excessive fat accumulation is distinguished in children with obesity. Imbalance between energy intake and expenditure is the root cause (3).

Described as simple, economical, safe, and feasible characteristic, exercise has been considered as a favorable measure to prevent and treat overweight/obesity in children and adolescents. Although the mechanisms of exercise-induced beneficial effects are not fully understood, convincing conclusions have been drawn that exercise can promote the browning effect of white adipose tissue (WAT) (4), improve insulin sensitivity, and regulate metabolism of glucose and lipids in obese bodies (5). However, the molecular mechanisms of WAT browning and reversing insulin resistance are less clear. Moreover, investigation is lacking on the difference between obese individuals and non-obese individuals in the responsiveness to exercise-mediated browning of WAT (6).

The mechanisms on exercise-mediated beneficial effects remain to be portrayed in-depth. Exosomes emerge as a type of extracellular vesicles carrying a large number of biological information molecules (e.g., protein, DNA, RNA, mRNA, and miRNA), which act in regulating intercellular communication and contribute to various physiological and pathological processes (7). A growing body of evidence indicates that circulating miRNA levels are good biomarkers for quantifying the physiological impacts of dietary or lifestyle intervention studies (8). In addition, miRNA expression in circulation varies widely between subjects who exercise or those who do not exercise (9). People have recognized that miRNA plays an important role in regulating energy homeostasis and WAT browning, affecting glucose and lipid metabolisms and insulin sensitivity (10). Among them, miR-27a exhibits a prominent role. miR-27a is highly expressed in adipose tissue (11). In a cold exposure study in mice, miR-27a was downregulated in subcutaneous WAT; meanwhile, researchers also showed its direct effect on PPAR-γ during brown adipogenesis of primary preadipocytes *in vitro*. The results pointed to miR-27a as a central upstream regulator of the transcriptional network involved in brown adipogenesis after cold exposure (12). Adipocyte-derived extracellular miR-27a was proposed as a messenger between adipose tissue and other tissues (13). Yu Y et al. showed that the serum miR-27a level correlated positively with obesity and insulin resistance in obese children, and adipocyte-derived exosomal miR-27a could result in insulin resistance in skeletal muscles by targeting PPAR-γ (14).

Skeletal muscles serve as an important exercise and energy metabolism organ. The resistance state in skeletal muscles can significantly affect systemic glucose homeostasis and insulin sensitivity (15). It is thought that skeletal muscle insulin resistance even precedes hepatic insulin resistance in humans with metabolic syndrome (16). In addition, miRNA expression in circulation varies widely between subjects who exercise or do not exercise (9).

Thus, the present study aims to explore the possible cause of exercise affecting WAT browning and skeletal muscle insulin resistance from the perspective of exosomal miRNA. Juvenile mice were fed with a high-fat diet or basal diet and took voluntary exercise at the same time to simulate the process of childhood obesity. The responses in obese and non-obese groups were compared, as well as exercise and non-exercise groups; particularly, the alteration of exosomal miR-27a and PPAR-γ was evaluated *in vivo*.

## Materials and Methods

### Animals

In total, 60 first-weaned C57BL/6 male mice were purchased from Beijing Huafukang Biotechnology Co., Ltd. (experimental animal production license number: SCXK (Beijing) 2019-0008). After 1 week of adaptive feeding, the mice were randomly divided into two dietary groups (*n* = 30/group) and fed with either a basal diet (13% fat) or high-fat diet (60% fat, D12492, Research Diets, China). Mice in each dietary group were then randomized into quiet group (untrained), 1 h exercise group, and 2 h exercise group. Consequently, six groups (*n* = 10/group) were analyzed during this study: basal quiet group (CON-S), basal 1 h exercise group (CON-PA1), basal 2 h exercise group (CON-PA2), high-fat quiet group (HFD-S), high-fat 1 h exercise group (HFD-PA1), and high-fat 2 h exercise group (HFD-PA2). Dietary and exercise interventions were delivered simultaneously for 10 weeks. All mice were housed in standard cages, and their food intake was recorded weekly.

### Exercise Protocol

Exercise intervention was carried out through non-weight-bearing swimming training. The exercise program was based on the mouse swimming exercise load standard in “Resource Book for the Design of Animal Exercise Protocols” published by the American Physiological Society in 2006 and referred to the relevant literature (17). CON-PA1 and HFD-PA1 groups swam 2 × 30 min every day with a 5 min interval; CON-PA2 and HFD-PA2 groups swam 4 × 30 min every day with a 5 min interval. In the 1st week, all mice in the exercise groups performed adaptive training. Intensity was 20 min/day in the first 3 days and then increased gradually every day. From the 2nd week, the training mice swam for 60 min or 120 min per day, respectively, for 5 days, with a 2 day interval for 10 weeks. Swimming training was carried out in a swimming tank of 120 × 80 × 80 cm, and the water temperature was controlled at 30 ± 2°C.

### Body Composition Measurements and Tissue Collection

Fat and lean masses were measured by using a Bruker Minispec LF50 (Bruker, Germany). After the mice were anesthetized with 3% sodium pentobarbital, one eyeball was enucleated, and blood was collected. The serum was centrifuged at 1356 × *g* for 15 min at 4°C. After the mice were killed, the subcutaneous and visceral (epididymal and perirenal) WAT and quadriceps muscle were isolated. Fat coefficients were calculated, as previously described (18).

### Insulin Tolerance Tests

The blood from the tail vein of the mice was dropped on the blood glucose test strip and measured by using a blood glucose meter. For the OGTT, after fasting for 12 h, the mice were given glucose (2 g/kg) by intragastric administration, and the blood glucose values were measured at 0, 15, 30, 60, 90, and 120 min. For the ITT, after fasting for 2 h, the mice were injected with insulin (1 U/kg), and the blood glucose values of the mice were measured at 0, 15, 30, 45, and 60 min (19). All values were recorded to draw curves, and the area under the curve (AUC) and ITT-AUC were calculated.

### Serum Preparation and Parameter Tests

Serum triglyceride (TG), total cholesterol (TC), low-density lipoprotein cholesterol (LDL-C), and high-density lipoprotein cholesterol (HDL-C) levels were measured using specific reagent kits (Nanjing Jiancheng, China). Insulin was measured by ELISA (Cusabio, Wuhan, China). HOMA-IR was calculated as the fasting insulin level (mU/L) × fasting blood glucose level (mmol/L)/22.5.

### Histology

White adipose tissue and skeletal muscle tissue were fixed with 4% paraformaldehyde for 24-48 h. Paraffin sections were stained with hematoxylin and eosin (H&E). Panoramic image acquisition was performed using a scanner, and Image-Pro Plus 6.0 software was used for analysis.

### Isolation of Serum Exosomes

Serum exosomes were isolated according to the instruction of Ribo™ exosome isolation reagent (Ribo, Guangzhou, China).

### Transmission Electron Microscopy to Identify Exosomes

The exosome PBS suspension was taken on the sample-carrying copper plate and allowed it to stand for 4 min; excess liquid was wiped off with a filter paper; negative staining with phosphotungstic acid was performed for 2 min; a filter paper was used to absorb excess water; its size and shape were observed under a transmission electron microscope; and pictures were collected.

### Gene Expression

Total RNA was extracted with TRIzol (TaKaRa, Japan). RNA purity and concentrations were determined with a Nanodrop 2000 spectrophotometer (Thermo, United States). The concentrations generally ranged from 500 to 700 ng/ul. The A260/280 absorbance ratio of 1.8:2.1 was used to assess the purity of RNA and DNA preparations. Totally, 2 μg RNA was extracted for DNA digestion and reverse transcription. Gene expression was analyzed by TB Green real-time quantitative PCR (qPCR) (TaKaRa, Japan). PCR amplification was performed using a QuantStudio6 Flex Real-time Quantitative PCR system (Applied Biosystems, United States) as follows: 95°C for 30 s, one cycle; 95°C for 5 s, 60°C for 34 s, 40 cycles; 95°C for 15 s, 60°C for 1 min, and 95°C for 15 s, one cycle. The level of mRNAs and miRNAs was normalized to the levels of β-actin or U6 exanimated in each sample using the 2^–ΔΔCT^ method. All primer sequences are listed in Table 1.

**TABLE 1:** Primer sequences of RT-PCR.

RNA		Sequences(5′-3′)
β-actin	Forward	GGCTGTATTCCCCTCCATCG
	Reverse	CCAGTTGGTAACAATGCCATGT
PPAR-γ	Forward	GACGCGGAAGAAGAGACCTG
	Reverse	GTGTGACTTCTCCTCAGCCC
PRDM16	Forward	AGGATTGCGAGCGGATGTT
	Reverse	GGCGGATGAGGTTGGACTT
PGC-1α	Forward	TGTGCTGCTCTGGTTGGT
	Reverse	GCCTCATTCTCTTCATCTATCTTCT
UCP1	Forward	CAAGAGGAAGGGACGCTCAC
	Reverse	AGTTGTCGGGTTCACCATCC
IRS-1	Forward	GGGACTGGGGGAGACATAGT
	Reverse	CTGGAGGAAGCTCGCAGAAA
GLUT-4	Forward	GCCCGGACCCTATACCCTAT
	Reverse	GGGTTCCCCATCGTCAGAG
AKT	Forward	TAGGCCCAGTCGCCCG
	Reverse	TCGTTCATGGTATCCGTGGC
miR-27a	Forward	GGTTCACAGTGGCTAAGT
	Reverse	CAGTGCGTGTCGTGGAGT
U6	Forward	CTCGCTTCGGCAGCACAT
	Reverse	AAATATGGAACGCTTCACG

### Dual-Luciferase Reporter Assay

The sequences of 3′-UTR of PPAR-γ were chemically synthesized and introduced into the luciferase reporter vector to construct the wild-type (WT) luciferase reporter plasmids, and the seed regions of miR-27a-3p in the 3′-UTR of PPAR-γ were mutated to construct mutant (Mut) luciferase reporter plasmids. 293T cells were co-transfected with luciferase reporter plasmids and miRNA mimics or inhibitors. After 48 h, the cells were collected, and dual-luciferase activity was measured using the Dual-Luciferase Reporter Assay (Ribo, Guangzhou, China) according to the manufacturer’s instructions.

### Western Blot Analysis

For the analysis, 20 μg soluble protein was isolated with 7.5/10% SDS-PAGE gel and transferred to a polyvinylidene fluoride membrane, enclosed with 5% skim milk. The protein was incubated overnight with a primary antibody at 4°C and then incubated with a secondary antibody at room temperature for 1 h. The target protein was normalized according to the β-actin protein content.

### Statistical Analyses

Statistical analyses were performed by SPSS software. The data are expressed as mean ± SD. Student’s *t*-test was used for comparison between the two groups, and one-way ANOVA or two-factor ANOVA was used for comparison between three or more groups. *P*-values < 0.05 were considered statistically significant.

## Results

### Exercise Hindered High-Fat Diet-Induced Body Fat Development in Growing Mice

**FIGURE 1:**
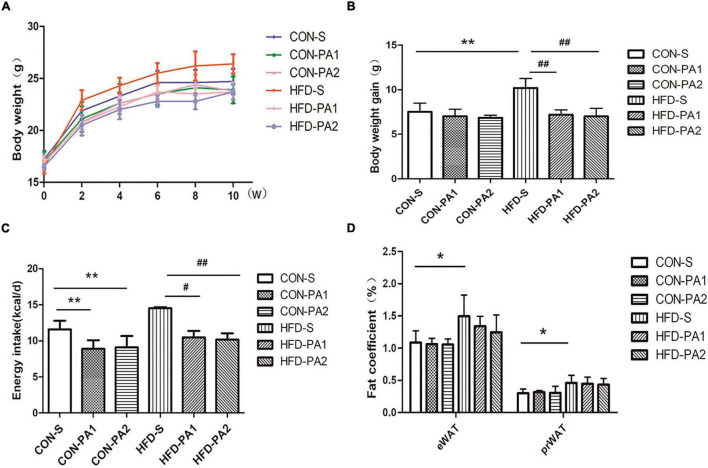
Effects of exercise on body weight, body weight gain, energy intake, and visceral fat in juvenile mice. **(A)** Bodyweight change. **(B)** Comparison of body weight gain. **(C)** Comparison of energy intake. **(D)** Comparison of epididymal and perirenal fat coefficients. Compared with the CON-S group, *P < 0.05 and **P < 0.01; compared with the HFD-S group, *^#^P* < 0.05 and ^##^*P* < 0.01.

To observe the anti-obesity effect of exercise, juvenile mice were fed with a basal diet (CON) or high-fat diet (HFD) and took swimming exercise simultaneously for 10 weeks. Compared with the CON-S group, the HFD-S group mice were obese, which had higher body weight, more body weight gain, a higher body fat ratio, and a visceral fat coefficient (Figures 1A,B,D). Exercise training significantly reduced the effect of body weight gain of HFD feeding (Figures 1A,B). Energy intake of exercise mice decreased compared with non-exercise mice (Figure 1C). The total body fat mass reduced in HFD-PA1 and HFD-PA2 groups, but the body fat mass percentage changes were not obvious in these exercise groups. The effects of exercise on lean body mass were not obvious in the two dietary treatments (Table 2).

**TABLE 2:** Comparison of body composition at termination.

Group	Body fat		Lean body	
	Mass (g)	Percentage (%)	Mass (g)	Percentage (%)
CON-S	0.87 ± 0.20	3.36 ± 0.72	15.71 ± 1.29	60.07 ± 1.81
CON-PA1	0.73 ± 0.17	3.05 ± 0.65	14.64 ± 2.13	61.76 ± 6.33
CON-PA2	0.72 ± 0.13	2.86 ± 0.50	15.62 ± 1.67	68.82 ± 7.05
HFD-S	1.37 ± 0.23**	5.44 ± 1.10**	13.65 ± 1.24**	53.78 ± 2.40**
HFD-PA1	1.08 ± 0.33^#^	4.89 ± 1.20	12.47 ± 1.33	54.78 ± 4.78
HFD-PA2	1.09 ± 0.15^#^	4.66 ± 0.45	12.92 ± 1.01	54.81 ± 2.92

Compared with the CON-S group, *P < 0.05 and **P < 0.01; compared with the HFD-S group, ^#^P < 0.05 and ^##^P < 0.01.

**FIGURE 2:**
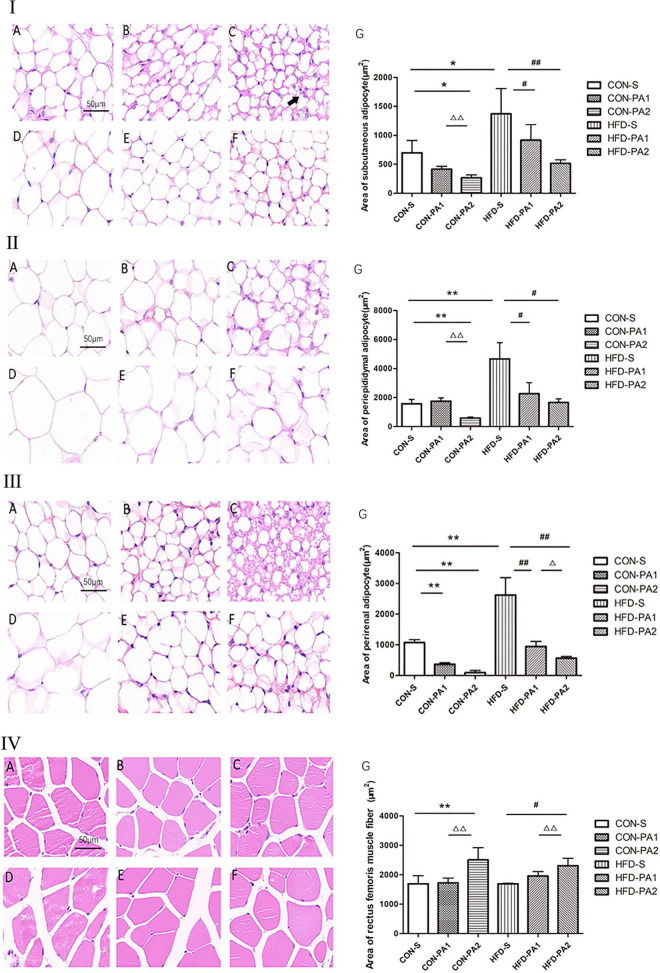
HE staining analysis of adipose tissue and skeletal muscles of mice. **(I)** Subcutaneous adipocytes. **(II)** Epididymal adipocytes. **(III)** Perirenal adipocytes. **(IV)** Skeletal muscle cells. (A) CON-S group. (B) CON-PA1 group. (C) CON-PA2 group. (D) HFD-S group. (E) HFD-PA1 group. (F) HFD-PA2 group. (G) Area comparison of adipocytes and skeletal muscle cells in each group. Compared with the CON-S group, *P < 0.05 and **P < 0.01; compared with the HFD-S group, *^#^P* < 0.05 and ^##^*P* < 0.01; comparison between exercise groups (CON-PA1 vs. CON-PA2, HFD-PA1 vs. HFD-PA2), ^Δ^*P* < 0.05 and ^ΔΔ^*P* < 0.01.

In addition, obvious morphological changes were seen in both CON and HFD exercise mice. Compared with the CON-S group, the adipocyte sizes of exercise mice (CON-PA1 and CON-PA2 groups) were smaller, while the cross-sectional area of skeletal muscle fibers in the HFD-PA2 group was significantly larger. Compared with the HFD-S group, the adipocyte sizes of exercise mice (HFD-PA1 and HFD-PA2 groups) were smaller, while the cross-sectional area of skeletal muscle fibers in the HFD-PA2 group was significantly larger (Figures 2I–IV).

### Exercise Modified Glucolipid Metabolism Parameters of Obese/Non-obese Growing Mice

**FIGURE 3:**
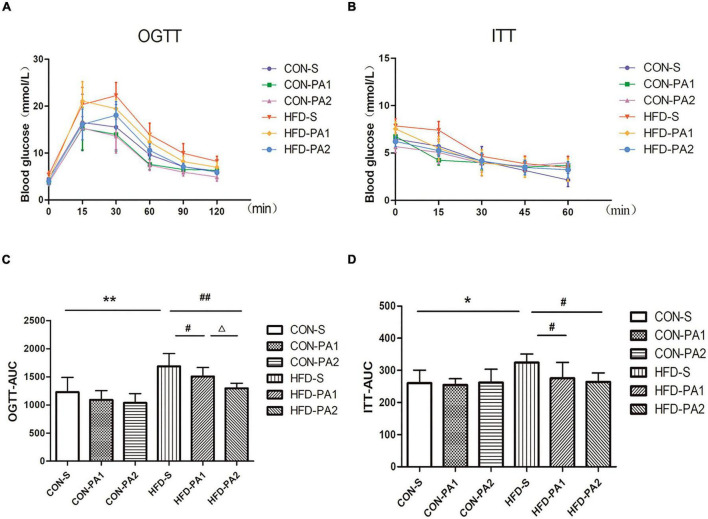
Glucose change curves of OGTT and ITT. **(A,C)** OGTT in mice after fasting for 12 h. Histograms represent the areas under the glucose curve. **(B,D)** ITT in mice after fasting for 2 h. Histograms represent the areas under the glucose curve. Compared with the CON-S group, *P < 0.05 and **P < 0.01; compared with the HFD-S group, *^#^P* < 0.05 and ^##^*P* < 0.01; comparison between exercise groups (CON-PA1 vs. CON-PA2 and HFD-PA1 vs. HFD-PA2), ^Δ^*P* < 0.05 and ^ΔΔ^*P* < 0.01.

To investigate the therapeutic effect of exercise on metabolic disorders, serum biochemical parameters related to lipid and glucose metabolisms were measured. Compared with the CON-S group, TG, TCHO, LDL-C, FPG, FINS concentrations, and HOMA-IR of the obese HFD-S group were significantly increased. In basal diet-fed groups, 2 h exercise significantly reduced the levels of TCHO and LDL-C and increased the level of HDL-C. In HFD-fed groups, 2 h exercise significantly reduced the levels of TG and TCHO and increased the level of HDL-C; 1 and 2 h exercise significantly reduced the levels of FPG, FINS, and HOMA-IR (Table 3). Since insulin resistance is highly positively correlated with the progression of obesity, OGTT and ITT were completed for evaluating whether exercise could improve the insulin sensitivity of obese mice. The results showed the OGTT-AUC and ITT-AUC were decreased in the HFD-PA1 and HFD-PA2 groups compared with HFD-S group (Figure 3).

**TABLE 3:** Serum biochemical parameter tests at termination.

	TG (mmol/L)	TCHO (mmol/L)	LDL-C (mmol/L)	HDL-C (mmol/L)	FPG (mmol/L)	FINS (mU/L)	HOMA-IR
CON-S	0.49 ± 0.06	3.32 ± 0.36	1.29 ± 0.23	2.66 ± 0.88	4.32 ± 0.59	15.37 ± 2.21	2.94 ± 0.53
CON-PA1	0.44 ± 0.04	3.13 ± 0.26	1.15 ± 0.16	2.89 ± 0.37	3.67 ± 0.60	14.34 ± 2.26	2.33 ± 0.49
CON-PA2	0.31 ± 0.04	3.01 ± 0.19*	1.02 ± 0.14*	3.41 ± 0.72*	3.65 ± 0.63	17.00 ± 3.20	2.81 ± 0.86
HFD-S	0.60 ± 0.07*	4.23 ± 0.31*	1.73 ± 0.27*	2.56 ± 0.08	5.73 ± 0.64**	19.48 ± 1.25**	4.95 ± 0.43**
HFD-PA1	0.43 ± 0.08	3.99 ± 0.33	1.62 ± 0.15	2.74 ± 0.47	4.35 ± 0.63^##^	18.63 ± 1.33^##^	3.60 ± 0.60^##^
HFD-PA2	0.38 ± 0.07^#^	3.67 ± 0.14^#^	1.46 ± 0.09	3.38 ± 0.52^#^	4.10 ± 0.80^##Δ^	15.79 ± 3.12^##^	2.79 ± 0.20^##ΔΔ^

TG, triglyceride; TCHO, total cholesterol; LDL-C, low-density lipoprotein cholesterol; HDL-C, high-density lipoprotein cholesterol; FPG, fasting plasma glucose; FINS, fasting serum insulin; HOMA-IR, homeostasis model assessment of insulin resistance. Compared with the CON-S group, *P < 0.05 and **P < 0.01; compared with the HFD-S group, ^#^P < 0.05 and ^##^P < 0.01; comparison between exercise groups (CON-PA1 vs. CON-PA2, HFD-PA1 vs. HFD-PA2), ^Δ^P < 0.05 and ^ΔΔ^P < 0.01.

### Exercise Reduced Serum Exosomal miR-27a Level in Obese Growing Mice and the Prediction Target Gene

**FIGURE 4:**
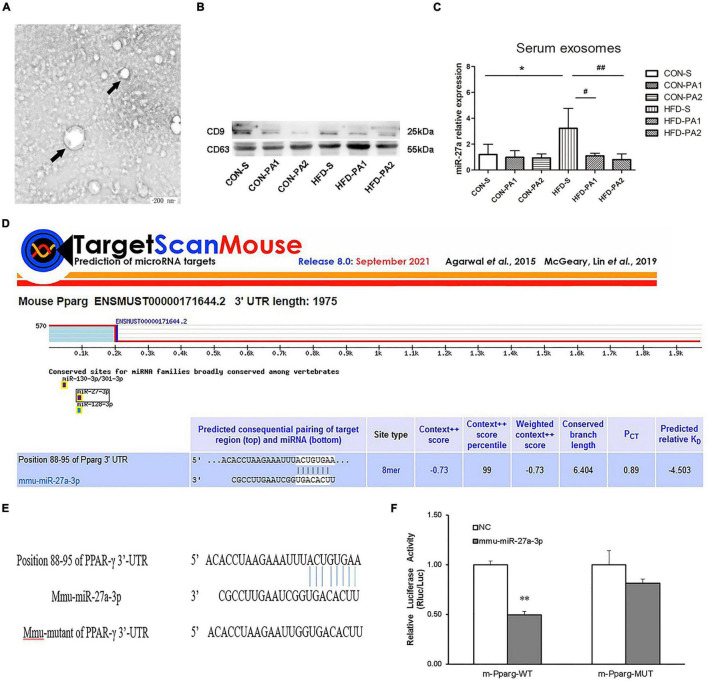
Serum exosome miR-27a level and target gene prediction. **(A)** TEM demonstrated that exosomes secreted by mice were round vesicles (bars = 200 nm). **(B)** Western blot detected the protein expressions of CD9 and CD63 in exosomes. **(C)** Expressions of exosomes miR-27a were measured by qRT-PCR. **(D)** Prediction of the target gene of miR-27a. **(E)** Schematic of the miR-27abinding site to PPAR-γ 3′-UTR. **(F)** Luciferase reporter assay indicated the binding of miR-27a with PPAR-γ. Compared with the CON-S group, *P < 0.05 and ^**^P < 0.01; compared with the HFD-S group, *^#^P* < 0.05 and ^##^*P* < 0.01; comparison between exercise groups (CON-PA1 vs. CON-PA2, HFD-PA1 vs. HFD-PA2).

Exosomes contain a large number of miRNAs. It has been reported that exercise leads to changes in the expression of miR-27a *in vivo* (20). Exosomes were extracted from mouse serum by using kits, and the marker proteins (CD9 and CD63) were detected by Western blot experiments (Figures 4A,B). We also observed that the expression of exosomal miR-27a in obese mice was increased significantly, while that was reduced significantly in exercise mice with HFD (Figure 4C). We predicted PPAR-γ as a potential target downstream of miR-27a by using TargetScan (Figure 4D). Luciferase reports have verified that the reported fluorescence of M PPARG MUT increased significantly compared with M PPAR-γ WT after co-transformation with MMU miR-27a-3p. These results suggest that MMU miR-27a-3p is likely to interact significantly with this site on the predicted M PPARG 3′UTR (Figures 4E,F).

### Exercise Stimulated White Adipose Tissue Browning

The browning of adipocytes plays a positive role in improving obesity, and subcutaneous WAT is the sensitive site. Results showed that obese mice’s expression of UCP1 was significantly reduced in the HFD-S group compared with the CON-S group. Exercise could upregulate the expression of UCP1, and the comparison between HFD-PA2 and HFD-S was significant, as well as CON-PA2 versus CON-S.

**FIGURE 5:**
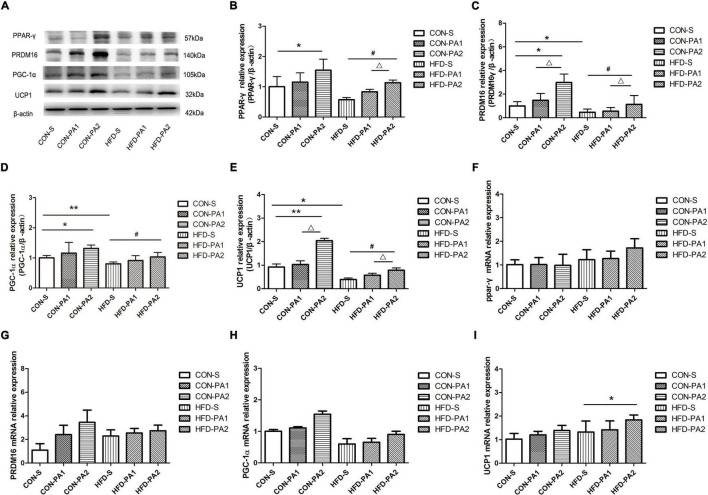
Expression levels of browning-related proteins and genes in mice WAT. **(A–E)** Western blot analysis of protein PPAR-γ, PRDM16, PGC-1α, and UCP1 in subcutaneous adipose tissues. **(F–I)** Relative expression analysis results of PRDM16, PPAR-γ, PGC-1α, and UCP1 genes, respectively. Compared with the CON-S group, *P < 0.05 and **P < 0.01; compared with the HFD-S group, *^#^P* < 0.05 and ^##^*P* < 0.01; comparison between exercise groups (CON-PA1 vs. CON-PA2, HFD-PA1 vs. HFD-PA2), ^Δ^*P* < 0.05 and ^ΔΔ^*P* < 0.01.

To investigate the effect of exercise on the browning of WAT, the expression levels of adipose tissue brown-related proteins and genes were also examined (21). We found that the expression levels of three main transcriptional regulators of classical brown adipose tissue (BAT) development, namely, PPAR-γ, PR domain containing 16 (PRDM16), and peroxisome proliferator-activated receptor γ coactivator 1α (PGC-1α), were upregulated in the exercise mice, regardless consuming a basal diet or HFD (Figure 5). Meanwhile, we observed a small number of cells similar to brown adipocytes in subcutaneous WAT of mice in the CON-PA2 group. These cells were small in size, their nucleus was located in the center, and there were many small fat droplets around them (Figures 2I–III).

### Exercise Activated IRS-1/Akt/GLUT-4 Signaling Pathway in Skeletal Muscle of Obese Growing Mice

**FIGURE 6:**
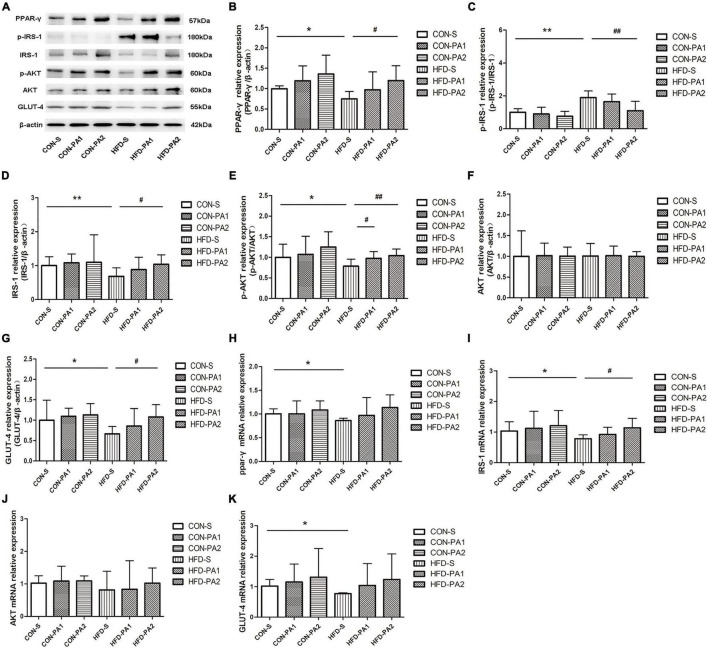
Expressions of glucose uptake-related proteins in skeletal muscles of mice. **(A–G)** Western blot analysis of proteins PPAR-γ, p-IRS-1, IRS-1, p-AKT, AKT, and GLUT-4 in skeletal muscles of mice. Compared with the CON-S group, *P < 0.05 and **P < 0.01; compared with the HFD-S group, *^#^P* < 0.05 and ^##^*P* < 0.01; comparison between exercise groups (CON-PA1 vs. CON-PA2, HFD-PA1 vs. HFD-PA2).

Skeletal muscle insulin resistance is mainly characterized by impaired insulin-dependent glucose uptake. In this research, WB and qPCR experiments were carried out to evaluate the expressions of PPAR-γ and key molecules for insulin-dependent glucose uptake. We found that expressions of PPAR-γ and glucose transporter type 4 (GLUT-4) were decreased in the HFD-S group compared with the CON-S group. However, their expressions were upregulated by exercise in the HFD-PA2 group compared with the HFD-S group. Key molecules and their phosphorylation in the insulin signaling pathway were measured. The phosphorylation of insulin receptor substrate-1 (IRS-1) on Ser307 was enhanced, and phosphorylation of protein kinase B (AKT) was inhibited in the HFD-S group compared with the CON-S group. However, swimming exercise significantly decreased the expression of p-IRS-1 and increased the expression of p-AKT in the HFD-PA2 group (Figure 6A–K).

## Discussion

In recent years, the incidence of overweight and obesity among children and adolescents has increased rapidly, which has become a prominent public health problem in the world (1). Overnutrition and insufficient exercise are the primary causes. Exercise intervention has been proved to be an effective way to control weight and treat childhood obesity (22). Swimming is often used in investigating the effect of exercise on adipose tissue and skeletal muscles (23, 24). In the present study, juvenile mice developed obese manifestation when fed with a high-fat diet continuously for 10 weeks, which stimulated the development process of childhood obesity. In PA1 and PA2 subgroups in each dietary treatment, the mice performed non-weight-bearing swimming training, which were applied to observe the different responses induced by exercise.

All mice completed the exercise programs without difficulty. Exercise hindered diet-induced obesity occurring in the development stage. In terms of physical outcomes, the HFD-S mice gained more body weight after 10 weeks of feeding than those in the CON-S group. Likewise, fat weight, body fat ratio, and epididymal and perirenal fat coefficients were significantly increased in the HFD-S group. Through the swimming training intervention, the fat mass of the exercising mice in the HFD-PA1 and HFD-PA2 groups was significantly less than that of non-exercising ones in the HFD-S group. Interestingly, the effects of exercise on body fat and lean body were not obvious in mice fed with a basal diet, and a similar phenomenon was also observed in a previous 10 week treadmill study in mice (25). Examining the mice in non-exercise groups (CON-S and HFD-S), the lean body mass of obese mice was smaller than that of the normal control; the consequence was probably due to fast growing of body fat induced by the HFD in young animals. Apart from this, the difference of lean body mass was low in each dietary group. These results indicated that exercise tended to favor regulation of body fat on diet-induced obesity.

Exercise-induced adaptive responses can be seen from cellular morphological changes. The size of the adipocyte volume is related to the intracellular lipid droplets. Adipocyte hypertrophy was observed in obese animals, which could be ameliorated by exercise, a similar effect also occurred in those mice on the basal diet. Bae JY et al. have substantiated that the catabolic rate of mice is higher than the anabolic rate after exercise, and lipid droplets are decomposed, thereby the volume of adipocytes is reduced (26). Exercise could also regulate skeletal muscle plasticity. The cross-sectional areas of muscle cells, especially in the 2 h exercise groups, were significantly larger than those in quiet groups either on the basal diet or HFD. Skeletal muscles are the material basis of body performance and physical activity, which can change its physical properties and composition in response to physiological demands. The influence of obesity on the intrinsic force-producing capacity of skeletal muscles is not completely resolved yet (27). Although contractile performance and constituent fibers of skeletal muscles were not measured in this study, the change in muscle fibers had been presented, which reflected high adaptable ability of skeletal muscles. Our results confirmed that for impeding the pandemic of childhood obesity, the duration of exercise in each session is crucial to mediate a certain beneficial effect on the skeletal muscle cell structure. In addition, interventions must be multifaceted, and we cannot emphasize the importance of diet, physical exercise, and developing healthy living habits in children too much at any time.

Obesity is a chronic metabolic disease which links to multiple factors and results from long-term imbalance between energy intake and consumption (3). Exercise can not only increase energy consumption and fat oxidation but also affect hormones that regulate appetite. As a result, energy intake is limited, and weight is controlled consequently (28). Although the hormones associated with regulating appetite were not included in measuring parameters in the present study, it was observed that food intake of exercising mice was less than the quiet ones, especially in the case of HFD feeding. The appetite of the mice under swimming training was influenced notably, which supported the opinion that higher habitual physical activity levels may better adjust body weight and body composition not only by increasing energy expenditure but also by reducing food intake (28, 29).

As is well known, long-term HFD feeding can lead to interference of glucolipid metabolism and increase the risk of various metabolic diseases. A large number of literature studies have been well documented that aerobic exercise can ameliorate obesity, modify the lipid profile, and improve insulin sensitivity (30–32). Our results showed that the serum levels of TCHO, TG, and LDL-C in the HFD-diet quiet group were significantly higher than those in the basal-diet quiet group, which indicated that the HFD caused lipid metabolism disorder in obese mice during the development stage. The influence of exercise on the lipid profile was more noticeable in the mice swimming longer, which happened both in obese bodies and non-obese bodies. Recently, researchers also observed significant reduction of LDL, HDL, and TG in rats which underwent continuous swimming training (33). Similar results have been obtained in patients with dyslipidemia, and serum LDL-C, intermediate-density lipoprotein, and very low-density lipoprotein levels were significantly reduced after 16 weeks of aerobic exercise (34). Combined with the results of adipose tissue morphological sections, we are convinced that a HFD may more easily cause body fat accumulation and hyperlipidemia, and non-weight-bearing swimming for 10 weeks can effectively improve dyslipidemia and facilitate weight control.

The high-fat diet and lack of exercise are considered as risk factors for insulin resistance (35), which is usually associated with impaired glucose metabolism (36). In this study, FPG, FINS, and HOMA-IR indexes were used to determine whether insulin resistance occurred in the HFD-fed mice, and the OGTT and ITT were used for further verification. The levels of FPG, FINS, HMOA-IR index, and fasting OGTT and ITT were significantly higher in the HFD-S group than in the CON-S group, which indicated that system insulin resistance had occurred in the non-exercise HFD-S group. Excitedly, swimming training altered the glucose tolerance test and improved insulin sensitivity in the 1 h and 2 h exercise HFD-S groups. Similar results were also obtained in several previous studies (37–39). Insulin resistance induced by the HFD is mainly related to oxidation of the glucose–fatty acid cycle (40, 41). Sandu O et al. demonstrated that fatty diets contain pro-oxidation and pro-inflammatory compounds that are associated with impaired insulin sensitivity (42). To the best of our knowledge, the mechanism of development of insulin resistance is unclear; it has been generally accepted that the HFD is responsible for insulin resistance, decreased insulin-stimulated glycolysis, and glycogen synthesis (40–43). The induction of insulin resistance in skeletal muscles is a key phenomenon, and impairments in insulin signaling in this tissue directly contribute to hyperglycemia, which has been confirmed by the following results of insulin signaling pathway experiment. Although the benefits of exercise for insulin resistance have not been clarified whether it is the exercise itself or the weight loss that has resulted from the exercise (5), our research presents a promising picture for the benefits of exercise in ameliorating metabolic disorders involved in HFD-induced obesity in the developing stage.

As the largest energy reservoir in the body, adipose tissues govern the physical function of energy metabolism and exert a key role in maintaining energy balance (44). There are three distinct types of adipose tissue in rodents and humans: BAT, WAT, and beige adipose tissue. Each adipose tissue or adipocyte type has distinct morphological features and physiological functions (45). In rodents, brown adipocytes could be induced by a variety of stimuli, including cold exposure, drug, and exercise (4, 46, 47). In response to these stimuli, WAT browns and energy expenditure is promoted. Therefore, converting WAT to BAT, and activating and expanding BAT are the most important targets for combating obesity and metabolic diseases.

Researchers have examined the role of exercise-induced adaptations to adipose tissue, and an important change in rodents is the increase in “beiging,” which occurs in the subcutaneous depot (23). It has been reported that exercise caused subcutaneous white adipocytes of HFD-induced obese animals to display beige adipocyte characteristics, which included abundant multilocular adipocytes and upregulation of the beige fat-specific gene UCP1, whereas visceral fat appeared to be resistant to stimulation inducing the browning switch (48, 49). Xu X et al. found that the expression of the beige fat-specific gene UCP1 in WAT was upregulated after 8 weeks of treadmill exercise in the mice, regardless of whether they were fed with a HFD or basal diet (50). Another experiment showed that UCP1 and PRDM16 were significantly upregulated in subcutaneous WAT of adult mice after 11 days of autonomous wheel running training with a basal diet. Several studies also reported brown fat-like development in swimming training mice, and the expression of browning markers in WAT depots, such as UCP1 and PGC-1α, was significantly increased (51–53). In the current study, the juvenile mice performed non-weight-bearing swimming exercise for 10 weeks. As previous studies reported, subcutaneous WAT is sensitive to exercise stimulation, and we observed browning-like cells emerged in H&E-stained sections of the CON-PA2 group. Although a cellular morphology change was seen only in the basal diet-fed mice, the biomarker of brown adipocytes, namely, UCP1, and adipose tissue brown-related proteins and genes were upregulated both in the HFD-PA2 and CON-PA2 groups. Our results indicated that adaption to exercise of HFD-induced obese WAT lagged behind normal WAT. In addition, the morphological structure and molecular alteration occurred in 2 h exercise groups, which suggested exercise duration was essential for effective physical response. The exercise intervention was delivered for 10 weeks in the current experiment. It does not rule out obvious physical adaption to exercise occurring in the 1 h exercise session in some cases, such as a prolonged exercise period and appropriate increase in exercise intension.

Circulating miRNA levels might be a promising biomarker for monitoring the impact of lifestyle or dietary interventions (8). Extracellular miRNAs are enclosed in vesicles. Exosomes are one of the main forms; they are highly sensitive to exercise stress, which enter the circulation or tissues faster than adrenaline and other hormones to maintain the homeostasis of the intracellular environment and participate in the regulation of various physiological functions, such as body metabolism (54). Since exercise can trigger alteration of the exosomal profile in the circulation, a possible mechanism for the systemic benefits of exercise has been propagated (55). It was reported that miR-27a was highly expressed in sera of obese individuals with prediabetes and T2DM (56). Another research found that serum levels of miR-27a were positively associated with obesity and insulin resistance in children and mice with obesity, and the researcher speculated that miR-27a might be another modulator of obesity-associated insulin resistance (14). However, exercise-induced changes in circulatory miR-27a have received little attention. In this study, we found a significantly high expression level of serum exosomal miR-27a in obese mice, and swimming exercise decreased the expression level in the HFD-fed mice. Biological information technology was used to predict the target genes of miR-27a, and the results showed that the 3′-untranslated region of PPAR-γ was the direct target of miR-27a. At the same time, real-time PCR and Western blot were used for verification. In agreement with Chen T et al.’s study (57), our results suggested that PPAR-γ could be negatively regulated by miR-27a *in vivo*.

For confirming the findings of morphological changes of subcutaneous WAT, we detected three main transcriptional regulators of classical BAT development. PPAR-γ is the obligatory transcription factor indispensable for the differentiation and survival of both white and brown adipocytes (58). During adipose formation, PPAR-γ can induce the expression of UCP1, a hallmark of brown fat-like phenotype (59). In both mouse and human WATs, it has been confirmed that treatment with PPAR-γ activator rosiglitazone could induce the expression of UCP1 (58, 60). PRDM16 was first discovered and reported as a zinc finger protein that is especially expressed in BAT, which is required and sufficient to promote brown adipogenesis in WAT (61, 62). PGC-1α is named after its role as a coactivator of PPAR-γ, needed for BAT thermogenesis but not differentiation (63). In this study, we found that the protein expression levels of PPAR-γ, PRDM16, and UCP1 in subcutaneous WAT of mice in the 2 h exercise group were significantly higher than those in the quiet group, regardless of the dietary treatment. These results suggest that 2 h swimming exercise can decrease the expression of circulating exosome miR-27a connected with HFD-induced obesity and promote the browning of WAT in growing mice.

Occupying approximately 40% of the total body mass, skeletal muscles account for more than 70% of the whole body glucose disposal (64). Obesity plays a key role in the onset and progression of insulin resistance, which is a reversible condition but if untreated, leads to type 2 diabetes alongside. Insulin binds to insulin receptors and initiates the intracellular cascade events. The IRS-1/Akt/GLUT-4 pathway is the most investigated mechanism involved in disease development. In insulin signaling, IRS-1 is the most important insulin receptor substrate protein isoform in muscles. The phosphorylation of IRS-1 on tyrosine residues is required for insulin-stimulated responses, but increased phosphorylation of specific serine residues can render IRS-1 inactive (65). Then, downstream factor AKT is phosphorylated and activated through phosphatidylinositol 3-kinase (PI3K). Phosphorylation of IRS-1 and AKT affects glucose uptake (66). GLUT-4, a member of the GLUT family, maintains glucose balance by moving to the plasma membrane and promoting the uptake and transport of glucose into the cells on the membrane when regulated by the IRS-1/PI3K/AKT signaling pathway (67). Muscle insulin resistance is due to disturbed phosphorylation and glucose transport (68). It has been confirmed that PPAR-γ is a transcriptional regulator of IRS-1 and GLUT-4 (69). Skeletal muscles that are insulin resistant do not respond to insulin, which causes impairment in the insulin signaling pathway and, as a result, a reduction in GLUT-4 levels (70). Therefore, we studied whether exercise activates the insulin signaling pathway, upregulates GLUT-4 expression, and eventually improves skeletal muscle insulin resistance. Compared with the CON-S group, we found that the expression of PPAR-γ, p-AKT, and GLUT-4 was significantly downregulated; p-IRS-1 (Ser307) was significantly upregulated, which indicated that the IRS-1/Akt/GLUT-4 signaling pathway was diminished in obese growing mice induced by the HFD. However, 2 h exercise modulated the expression of transcriptional regulators PPAR-γ and GLUT-4, and phosphorylation of key signaling molecules of the insulin effect. Our results showed that exercise plays an improved role in the phosphorylation of IRS1 and AKT and leads to the modulation of GLUT-4 expression that would increase insulin-dependent glucose uptake and storage in skeletal muscles.

## Conclusion

In summary, juvenile mice developed obesity manifestation when fed with a HFD continuously, which is helpful to simulate the development process of childhood obesity. We demonstrated that the HFD more easily caused body fat accumulation. Non-weight-bearing swimming exercise for 10 weeks could effectively facilitate weight control and tend to favor the regulation of body fat on diet-induced obesity. Concerning the morphological observation of adipocytes and skeletal muscle cells, the changes of HFD-induced obese mice were consist with the normal control. Exercise modified glucolipid metabolism parameters differently in the obese/non-obese groups, and the changes in the 2 h exercise mice were not consistent, as well as 1 h exercise mice. The expression level of serum exosomal miR-27a was significantly high in obese mice, and swimming exercise decreased the expression level in the HFD-fed mice. Bioinformatics analysis and dual-luciferase reporter assay showed that miR-27a targeted PPAR-γ. Although UCP1 and adipose tissue brown-related proteins and genes were upregulated both in the HFD-PA2 and CON-PA2 mice, browning-like cells were seen only in H&E-stained sections of the CON-PA2 mice. Exercise activated the IRS-1/Akt/GLUT-4 signaling pathway in skeletal muscles of the HFD-PA2 group. Taken together, our findings implicated that exercise-induced beneficial effects are associated with the exercise duration, and the response of obese and non-obese bodies is different. Exosomal miR-27a might be a crucial node for the process of exercise-induced browning of WAT and improving skeletal muscle insulin sensitivity. However, future *in vitro* studies are required to validate the effects of miR-27a on adipocytes and skeletal muscle cells.

## Data Availability Statement

The original contributions presented in the study are included in the article/supplementary material, further inquiries can be directed to the corresponding author.

## Ethics Statement

The animal study was reviewed and approved by the Laboratory Animal Welfare and Ethics Committee of China Medical University.

## Author Contributions

DW and XZ performed the majority of the experiments and wrote the manuscript. YL drafted the part of the manuscript. LJ, LZ, and HJ contributed to the study design and technological support. LZ and WW helped with the data analysis. YB designed and supervised the study and checked the final manuscript. All authors contributed to the manuscript and approved the submitted version.

## Conflict of Interest

The authors declare that the research was conducted in the absence of any commercial or financial relationships that could be construed as a potential conflict of interest.

## Publisher’s Note

All claims expressed in this article are solely those of the authors and do not necessarily represent those of their affiliated organizations, or those of the publisher, the editors and the reviewers. Any product that may be evaluated in this article, or claim that may be made by its manufacturer, is not guaranteed or endorsed by the publisher.
